# Performance Evaluation of GPT-4o and o1-Preview Using the Certification Examination for the Japanese 'Operations Chief of Radiography With X-rays'

**DOI:** 10.7759/cureus.74262

**Published:** 2024-11-22

**Authors:** Hiroki Goto, Yoshioki Shiraishi, Seiji Okada

**Affiliations:** 1 Radioisotope and Tumor Pathobiology, Institute of Resource Development and Analysis, Kumamoto University, Kumamoto, JPN; 2 Radioisotope Center, Institute of Resource Development and Analysis, Kumamoto University, Kumamoto, JPN; 3 Hematopoiesis, Joint Research Center for Human Retrovirus Infection, Kumamoto University, Kumamoto, JPN

**Keywords:** artificial intelligence (ai), gpt-4o, large language model, o1-preview, x-ray safety management and protection

## Abstract

Purpose

The purpose of this study was to assess the ability of large language models (LLMs) to comprehend the safety management, protection methods, and proper handling of X-rays according to laws and regulations. We evaluated the performance of GPT-4o (OpenAI, San Francisco, CA, USA) and o1-preview (OpenAI) using questions from the 'Operations Chief of Radiography With X-rays' certification examination in Japan.

Methods

This study engaged GPT-4o and o1-preview in responding to questions from this Japanese certification examination for 'Operations Chief of Radiography With X-rays'. A total of four sets of exams published from April 2023 to October 2024 were used. The accuracy of each model was evaluated across the subjects, including knowledge about the control of X-rays, relevant laws and regulations, knowledge about the measurement of X-rays, and knowledge about the effects of X-rays on organisms. The results were compared between the two models, excluding graphical questions due to o1-preview's inability to interpret images.

Results

The overall accuracy rates of GPT-4o and o1-preview ranged from 57.5% to 70.0% and from 71.1% to 86.5%, respectively. The GPT-4o achieved passing accuracy rates in the subjects except for relevant laws and regulations. In contrast, o1-preview met the passing criteria across all four sets, despite graphical questions being excluded from scoring. The accuracy of all questions and relevant laws and regulations in o1-preview were significantly higher than those in GPT-4o (p = 0.03 for all questions and p = 0.03 for relevant laws and regulations, respectively). No significant differences in accuracy were found across the other subjects.

Conclusions

In the Japanese 'Operations Chief of Radiography With X-rays' certification examination, GPT-4o demonstrated a competent performance in the subjects except for relevant laws and regulations, while o1-preview showed a commendable performance across all subjects. When graphical questions were excluded from scoring, the performance of o1-preview surpassed that of GPT-4o in all questions and relevant laws and regulations.

## Introduction

Artificial intelligence (AI) has advanced remarkably across numerous fields, with particularly notable progress in medicine. These developments in AI are transforming medical research, diagnostics, treatment planning, and patient care, paving the way for innovations that enhance both the precision and accessibility of healthcare services [1-3]. Artificial intelligence chatbots use natural language processing (NLP) technology to understand human language and generate appropriate responses [4,5]. ChatGPT is a generative AI chatbot with advanced capabilities in NLP and multimodal tasks, powered by a large language model (LLM) developed by OpenAI (San Francisco, CA, USA) [6]. It has achieved impressive results on medical licensing exams worldwide, highlighting its capability to understand complicated medical knowledge [7-10]. ChatGPT is gaining attention in more specialized fields, including radiology [11-18].

In OpenAI's GPT series, GPT-4o is equipped with enhanced NLP capabilities, improving performance aspects such as output speed, answer quality, and supported languages, which were lacking in previous models [19]. Additionally, it seamlessly integrates and processes text, voice, and images in real-time. The latest version of the GPT-family model, o1-preview, which was trained with reinforcement learning and incorporates chain-of-thought, is suitable for solving complex logical problems and tasks, such as scientific analysis and mathematics [20].

Although AI chatbots are expected to play a role in medicine and specialized education, their application in training and supporting X-ray safety management and protection, an essential field for reducing hazards associated with the widespread medical use of radiation, remains largely unexplored. Evaluating the extent to which AI chatbots appropriately respond to X-ray safety management and protection holds academic significance, as it contributes to enhancing the quality of X-ray safety education and provides insights that drive the development of more specialized AI technologies.

An 'Operations Chief of Radiography With X-rays' works in various locations where X-ray equipment is used, such as research institutes, industrial settings, and educational facilities. Their role includes ensuring radiation safety, managing exposure levels, conducting regular equipment checks, and providing radiation protection guidance to maintain a safe working environment. The Japanese Industrial Safety and Health Act administers the 'Operations Chief of Radiography With X-rays' certification examination and issues certifications to those who pass. When performing tasks that involve the use of X-ray devices (excluding medical devices or devices with a rated tube voltage of 1,000 kV or higher by peak value), it is necessary to appoint an 'Operations Chief of Radiography With X-rays' for each controlled area from among those who hold the certification. In this study, we assessed the ability of GPT-4o and o1-preview to answer questions from the 'Operations Chief of Radiography With X-rays' certification examination in Japan and analyzed the performance of these LLMs in solving the problems of X-ray safety management and protection.

## Materials and methods

The GPT-4o and o1-preview

The GPT-4o was released on May 13, 2024, as a model capable of handling text, audio, and image inputs and delivering real-time responses across these formats [19]. OpenAI released o1-preview, its latest model, on September 12, 2024. Unlike previous models, o1-preview takes a moment to 'think' before responding, enhancing its performance in fields that require deep logical reasoning, such as mathematics, biology, and chemistry [20]. The GPT-4o is available at no cost, while ChatGPT Plus subscribers, paying $20 per month, receive five times the usage limit. Access to o1-preview is primarily available through ChatGPT Plus.

The Japanese 'Operations Chief of Radiography With X-rays' certification examination

The 'Operations Chief of Radiography With X-rays' is one of the operations chiefs under the Japanese Industrial Safety and Health Act. This position is appointed by the employer from individuals who have been granted this national certification by the Director of the Prefectural Labor Bureau. The certification examination for this qualification covers the following subjects: knowledge about the control of X-rays, relevant laws and regulations, knowledge about the measurement of X-rays, and knowledge about the effects of X-rays on organisms. The exam consists of 40 questions, with 10 questions for each subject. The scoring distribution is a total of 100 points, with 30 points for knowledge about the control of X-rays, 20 points for relevant laws and regulations, 25 points for knowledge about the measurement of X-rays, and 25 points for knowledge about the effects of X-rays on organisms. The exam duration is four hours. The passing criteria for the exam are a total score of 60% or more of the full score and a score of 40% or more in each subject. The pass rates from 2004 to 2023 have fluctuated between 35% and 63%. There were 5,746 candidates in 2023, of whom 2,578 passed, resulting in a pass rate of 44.9% [21]. The average annual scores for this exam are not officially disclosed. The exam questions are published once each in April and October. In this study, we used a total of four sets of exams published from April 2023 to October 2024. The questions for each set are available online [22]. All authors of this study hold the 'Operations Chief of Radiography With X-rays' certification.

Data input

The exam questions and their multiple-choice answers were used as they are originally written in Japanese. Instructions for using GPT-4o and o1-preview were also provided in Japanese. Since GPT-4o was able to recognize images but o1-preview was not, we input graphical questions into GPT-4o after confirming it understood the images, while we did not input graphical questions into o1-preview. The data input was performed in October 2024. The questions from the same year were answered by GPT-4o and o1-preview on the same day (see Appendix A).

Statistical analysis

The Mann-Whitney U test was used as a robust non-parametric alternative to the t-test, appropriate for comparing differences between two independent groups, especially with small sample sizes or non-normal data. A p-value below 0.05 was deemed to indicate statistical significance. Statistical analyses were conducted using GraphPad Prism software, version 9.2.0 (GraphPad Software Inc., San Diego, CA, USA).

## Results

The GPT-4o was able to generate answers for all 160 questions across four sets of the 'Operations Chief of Radiography With X-rays' certification examination (conducted in Japanese). However, o1-preview cannot recognize or interpret visual data. In this study, a total of 150 questions except the graphical ones were used to evaluate the performance of o1-preview. The GPT-4o was able to begin answering questions immediately for all tasks, whereas the o1-preview required a delay ranging from a few seconds to up to several tens of seconds before initiating responses for the questions we used. The evaluation of the responses by GPT-4o and o1-preview are presented in Table 1 and Table 2, respectively.

**Table 1:** The accuracy of GPT-4o's answers to the four sets of the 'Operations Chief of Radiography With X-rays' certification examination The month and year indicate when the exam questions were published. The percentage represents the accuracy rate.

Parameter	April 2023	October 2023	April 2024	October 2024
	Number of correct answers/total questions (%)			
All questions	23/40 (57.5)	23/40 (57.5)	25/40 (62.5)	28/40 (70.0)
Knowledge about the control of X-rays	6/10 (60.0)	8/10 (80.0)	7/10 (70.0)	9/10 (90.0)
Relevant laws and regulations	2/10 (20.0)	2/10 (20.0)	3/10 (30.0)	3/10 (30.0)
Knowledge about the measurement of X-rays	8/10 (80.0)	6/10 (60.0)	9/10 (90.0)	7/10 (70.0)
Knowledge about the effects of X-rays on organisms	7/10 (70.0)	7/10 (70.0)	6/10 (60.0)	9/10 (90.0)

**Table 2:** The accuracy of o1-preview's answers to the four sets of the 'Operations Chief of Radiography With X-rays' certification examination The month and year indicate when the exam questions were published. The percentage represents the accuracy rate. Graphical questions were excluded for the o1-preview analysis.

Parameter	April 2023	October 2023	April 2024	October 2024
	Number of correct answers/total questions (%)			
All questions	27/38 (71.1)	32/37 (86.5)	31/38 (81.6)	29/37 (78.4)
Knowledge about the control of X-rays	6/8 (75.0)	6/7 (85.7)	8/8 (100.0)	7/8 (87.5)
Relevant laws and regulations	4/10 (40.0)	7/10 (70.0)	6/10 (60.0)	5/10 (50.0)
Knowledge about the measurement of X-rays	8/10 (80.0)	9/10 (90.0)	8/10 (80.0)	8/9(88.9)
Knowledge about the effects of X-rays on organisms	9/10 (90.0)	10/10 (100.0)	9/10 (90.0)	9/10 (90.0)

**Figure 1:**
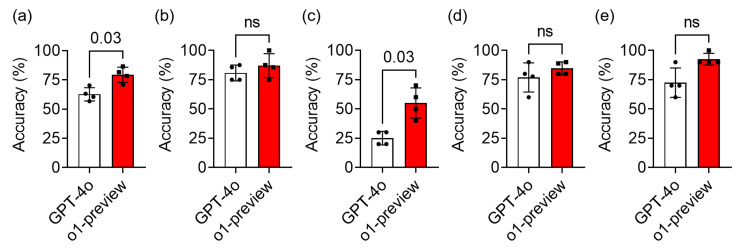
Comparison of the certification examination results between GPT-4o and o1-preview The accuracy of GPT-4o and o1-preview in all questions (a), knowledge about the control of X-rays (b), relevant laws and regulations (c), knowledge about the measurement of X-rays (d), and knowledge about the effects of X-rays on organisms (e) are shown. The data is shown as n = 4, %, and mean ± standard deviation. The numbers represent p-values. A p-value of less than 0.05 was considered to indicate statistical significance. ns: Not significant

The overall accuracy rates for GPT-4o and o1-preview varied between 57.5% to 70.0% and 71.1% to 86.5%, respectively, across four sets of exams published from April 2023 to October 2024. Considering the passing criteria, GPT-4o achieved sufficient scores in the subjects except for relevant laws and regulations (Table 1). In contrast, o1-preview attained passing scores in all four subjects (Table 2). Across the four exams taken in chronological order, GPT-4o scored 59.5, 60.5, 64.5, and 73 points, respectively. In comparison, the o1-preview's scores were 68.5, 79.5, 78.5, and 73.5 points, also listed chronologically. In the April 2023 exam, o1-preview barely met the minimum passing threshold of 40% accuracy on questions related to relevant laws and regulations. This trend of lower accuracy on questions related to relevant laws and regulations was observed in both models. However, it was more pronounced in GPT-4o, which was unable to pass any exams on this subject. Overall, GPT-4o failed to pass any of the four exam sets, primarily due to its low accuracy in relevant laws and regulations. In contrast, o1-preview passed all exams tested in this study, even with graphical questions omitted from its scoring due to its lack of image recognition capabilities. Figure 1 demonstrates the results of comparative analysis between GPT-4o and o1-preview.

To compare the performance of the two models, the accuracy of identical text-based questions was evaluated. In the o1-preview, the accuracy for all questions, as well as for relevant laws and regulations, was significantly higher than that of GPT-4o (p = 0.03 for all questions and p = 0.03 for relevant laws and regulations, respectively). There were no significant differences in accuracy across the other subjects. These results indicate that the performance of o1-preview surpassed that of GPT-4.0 in the accuracy of all text-based questions, especially those related to relevant laws and regulations in the 'Operations Chief of Radiography With X-rays' certification examination in Japan.

## Discussion

With demonstrated high accuracy in examinations and the ability to support complex decision-making, LLMs are positioned to enhance interdisciplinary knowledge and accessibility across various specialized fields [5,23]. In fact, LLMs like GPT-4 and GPT-4o have achieved high accuracy in professional exams, including the official radiology board exams [12-14]. However, the performance of LLMs, including GPT-4o and the latest o1-preview model, in understanding X-ray safety management and protection remains uncertain. In this study, we conducted a comparative assessment of GPT-4o and o1-preview on X-ray safety management and protection using the 'Operations Chief of Radiography With X-rays' certification examination in Japan.

As shown in Table 1, the performance of GPT-4o did not meet the passing criteria when taking all subjects into account. In contrast, although answers to the graphical questions were excluded, the o1-preview met the criteria and demonstrated sufficient performance to answer the questions in all subjects (Table 2). There were only two to three graphical questions in each set of exam questions from April 2023 to October 2024. Therefore, the impact of an inability to solve graphical questions on the score is considered minimal.

Parameters are the numerical values that allow LLMs to learn and retain language structures and contextual patterns, making them a crucial factor in determining the model's performance. The GPT-4o has significantly more parameters than the previous GPT-3.5 and GPT-4 models, resulting in improved accuracy and speed. With a chain-of-thought process, the o1-preview has the potential to handle complex logical tasks by applying advanced reasoning skills. In this study, the performance of both GPT-4o and o1-preview was not perfect, indicating that further improvement is needed. Hallucinations describe instances where LLMs produce incorrect or fabricated information [24,25]. Consequently, it is essential to critically assess LLM responses and verify information accuracy as needed.

In this study, there were relatively noticeable errors in responses to questions on relevant laws and regulations. Even in the o1-preview, accuracy in this subject ranged from 40.0% to 70.0%. We reported that GPT-4 and Gemini Advanced (Google DeepMind, London, GBR) met the passing standards in physics, chemistry, biology, and practical operations in the Japanese 'First-Class Radiation Protection Supervisor' examination; however, they fell short in the area of laws and regulations, likely due to frequent updates and the complexity of interpretation [15]. These recent findings are consistent with the results of this study, as the accuracy of GPT-4o and o1-preview on relevant laws and regulations tended to be lower compared with other subjects. Currently, it is crucial to review the precise legal language and consult the 'Operations Chief of Radiography With X-rays' for accurate interpretation of laws and regulations concerning X-rays.

Figure 1 shows that the o1-preview model outperformed GPT-4o in terms of test scores. These results suggest that incorporating technologies such as reinforcement learning and chain-of-thought processing into LLMs may enhance problem-solving and reasoning abilities in the specific field of X-ray safety management and protection. However, in terms of image recognition and response speed, GPT-4o appears to be superior. Therefore, it is essential to select LLMs while considering factors such as response immediacy, image recognition capabilities, and the complexity of logical questions.

This study has several limitations. First, the training data for GPT-4o and o1-preview may not fully reflect the specific content and scientific background of the 'Operations Chief of Radiography With X-rays' certification examination in Japan. Therefore, the AI’s performance may not be optimized for the unique characteristics of this exam. The LLMs may possess a general familiarity with legal overviews but are unlikely to have been trained on individual legal provisions, suggesting that machine learning incorporating specific provisions is necessary, which may also relate to the low accuracy in answering questions on relevant laws and regulations. Bias in the training data and an immature understanding of Japanese text in LLMs might also affect the performance. Second, these models do not always incorporate the latest knowledge, which may prevent them from responding accurately to questions involving recent updates. The timing of the model’s training may have affected its accuracy. Third, due to the ambiguous or partially correct responses that GPT models sometimes exhibit in their responses, setting clear evaluation criteria can be challenging, and measuring AI performance solely by accuracy rate has its limitations.

Given these limitations, future research should consider revising evaluation criteria to address these factors and developing LLMs that are better adapted to the specific nature of the exam. If AI's accuracy rates for this exam improve, it could be useful as a double-check for humans performing X-ray-related tasks and as a tool for learning about X-ray safety management and protection.

## Conclusions

The GPT-4o did not pass the Japanese 'Operations Chief of Radiography With X-rays' certification exam due to its low accuracy in the relevant laws and regulations section. The o1-preview on the other hand, met the required passing criteria in all four sets tested. The performance of the o1-preview surpassed that of GPT-4o in all questions, especially those related to relevant laws and regulations.

The GPT-4o is considered to have an advantage over the o1-preview in terms of image recognition and response speed. In contrast, the o1-preview can answer complicated logic questions in text-based questions. Depending on their respective characteristics, it is essential to apply GPT-4o and o1-preview appropriately in X-ray safety management and protection.

## Appendices

Appendix A

The responses of GPT-4o and o1-preview, together with the correct answers, are shown in Table 3.

**Table 3:** The responses of GPT-4o and o1-preview, along with the correct answers, in each set of the 'Operations Chief of Radiography With X-rays' certification examination. The exam consists of multiple-choice problems with one correct answer out of options 1 to 5, and the responses of GPT-4o and o1-preview, as well as the correct answers, are indicated by numbers. The month and year indicate when the exam questions were published. Not applicable (NA) is shown because o1-preview cannot handle graphical questions. The questions are divided into four parts, i.e., 1–10, 11–20, 21–30, and 31–40, covering knowledge about the control of X-rays, relevant laws and regulations, knowledge about the measurement of X-rays, and knowledge about the effects of X-rays on organisms, respectively. The exam questions, which are multiple-choice questions with options from 1 to 5, are available online [22].

Question no.	April 2023			October 2023			April 2024			October 2024		
	GPT-4o	o1-preview	Correct	GPT-4o	o1-preview	Correct	GPT-4o	o1-preview	Correct	GPT-4o	o1-preview	Correct
1	4	4	4	4	4	4	3	3	3	3	3	3
2	2	2	2	4	4	4	1	5	5	5	5	5
3	5	NA	2	3	3	3	1	1	1	1	1	1
4	5	5	5	5	5	5	4	4	4	3	3	3
5	1	1	4	5	NA	3	5	5	5	5	5	5
6	1	1	1	2	2	2	2	2	2	2	1	4
7	4	4	4	4	5	5	5	NA	4	5	5	5
8	5	5	2	3	NA	3	4	5	5	2	2	2
9	5	5	5	1	5	1	1	1	1	3	NA	3
10	2	NA	5	5	NA	5	5	NA	5	2	NA	2
11	1	1	5	2	5	5	2	3	3	4	2	2
12	4	3	2	5	5	5	4	1	1	2	2	3
13	5	5	5	3	2	2	2	2	4	3	2	3
14	5	3	2	1	1	4	4	4	4	2	3	3
15	3	4	1	3	3	3	5	5	5	1	1	1
16	2	3	3	2	5	5	3	2	2	5	1	4
17	1	3	2	3	4	4	1	2	1	1	2	2
18	3	3	3	3	4	2	2	3	3	4	5	4
19	4	4	5	5	1	1	2	4	5	3	5	4
20	3	1	1	1	2	3	1	3	4	5	2	2
21	4	4	4	4	4	4	4	4	4	4	4	4
22	5	5	5	4	2	2	5	5	5	2	2	2
23	1	3	2	1	1	1	3	3	3	1	1	1
24	2	2	2	5	5	5	3	5	3	5	5	5
25	4	4	3	1	2	2	5	2	5	4	4	4
26	1	1	1	5	5	4	4	4	4	2	2	2
27	2	2	2	1	1	1	4	4	4	5	3	3
28	3	3	3	1	1	1	4	4	4	5	5	5
29	3	3	3	5	5	5	5	3	3	4	NA	5
30	4	4	4	5	3	3	3	3	3	2	2	4
31	5	2	2	3	1	1	5	1	1	5	1	1
32	5	5	4	3	3	3	4	4	3	3	3	3
33	2	2	2	4	4	4	3	4	4	1	1	1
34	1	1	1	5	5	5	5	5	5	3	3	3
35	2	4	4	5	4	4	1	1	1	3	3	3
36	4	4	4	2	2	2	1	1	1	1	1	1
37	2	2	2	5	1	1	4	5	5	1	1	1
38	1	1	1	4	4	4	2	2	2	2	2	2
39	4	4	4	1	1	1	2	2	2	4	5	4
40	1	1	1	2	2	2	1	1	1	5	5	5
